# Validation of Hamdan intelligence scale in upper elementary grades using the Rasch model: exploratory study

**DOI:** 10.3389/fpsyg.2024.1407734

**Published:** 2024-08-30

**Authors:** Alaa Eldin A. Ayoub, Abdullah M. Aljughaiman, Mariam A. Alghawi, Ahmed Morsy, Ehab M. N. Omara, Ahmed M. Abdulla Alabbasi, Joseph S. Renzulli

**Affiliations:** ^1^Department of Gifted Education, Arabian Gulf University, Manama, Bahrain; ^2^Department of Educational Psychology, Aswan University, Aswan, Egypt; ^3^Department of Special Education, King Faisal University, Al-Ahsa, Saudi Arabia; ^4^Hamdan bin Rashid Foundation for Medical and Educational Sciences, Dubai, United Arab Emirates; ^5^Department of Psychology, Sultan Qaboos University, Muscat, Oman; ^6^Nega School of Education, University of Connecticut, Storrs, CT, United States

**Keywords:** intelligence, validity, reliability, unidimensionality, Rasch analysis, Hamdan Intelligence Scale

## Abstract

Hamdan Intelligence Scale (HIS) is the first intelligence scale that has been developed and normed in the United Arab Emirates (UAE). This study aimed to examine the refinement, validity, and reliability of HIS in upper elementary grades using the Rasch model. A total of 4,301 students (34.3% Male; 65.7% Female) from grade 4 to 6 (32.1% grade 4; 33.7% grade 5; 34.2% grade 6) were administered to the HIS. The confirmatory factor analysis was first conducted to verify the fitness of the one-factor model of the HIS. The results of validity showed strong correlation coefficients between the HIS and the Aurora-*g* battery (0.83) and the Raven’s Standard Progressive Matrices (RSPM; 0.86). Moreover, the results of the developmental trends demonstrated that raw scores of the HIS increase with age and grade relatively constantly across composite scores. Unidimensionality was confirmed through the Confirmatory Factor Analysis and Principal Component Analysis of Residuals (PCAR). The low eigenvalues of the first contrast were below 2, and additionally, the infit and outfit mean squares ranged from 0.88 to 1.14 and 0.84 to 1.14. Rasch’s person reliability result of 0.62 was acceptable reliability. The results provided strong support for the validity and reliability of using the Hamdan Intelligence Scale in the UAE environment.

## 1 Introduction

Intelligence quotient (IQ) tests are widely used around the world for many purposes (Coaley, 2010; Gottfredson and Saklofske, 2009), one of which is to identify gifted children (McIntosh et al., 2012). Educators can identify students with exceptional intellectual potential using these tests, which provide objective and standardized measurements of cognitive abilities (Pfeiffer, 2015). Moreover, IQ tests can tailor educational programs to meet students’ specific needs, ensuring that academic challenges and growth are appropriate to their needs (Borgonovi and Ferrara, 2020; Gibbons and Warne, 2019).

A high level of intelligence, as assessed by different IQ tests, is generally considered an important aspect of giftedness (Ayoub et al., 2022; Ziegler and Phillipson, 2012); therefore, IQ tests remain the most popular measure for identifying gifted learners. In the United States, for example, intellectual giftedness is included in 90 percent of the States’ definitions of giftedness (McClain and Pfeiffer, 2012) and 99.5% of school district definitions (Callahan et al., 2017). In approximately 32% of States, IQ tests are used to identify gifted students (McClain and Pfeiffer, 2012). IQ tests measure a person’s cognitive abilities, such as verbal comprehension, processing speed, perceptual organization, and working memory (Ganuthula and Sinha, 2019; Resing, 2004; Wechsler, 1997). Some of the benefits of using the IQ tests in identifying gifted students include (a) their objectivity and predictive validity (Nakano et al., 2016), (b) their ability to differentiate between high achievers and gifted students (Ayoub and Aljughaiman, 2016), (c) their ability to help in identifying twice-exceptional learners (Silverman, 2018), (d) their ability to help in identifying underachiever gifted students (Reis and McCoach, 2000; Ziegler and Stoeger, 2003), and (e) their ability to identify the relative strengths and weaknesses of gifted students and provide a basis for differentiating among them (Kaufman et al., 2016).

Due to their limitations, IQ tests should not determine giftedness solely (Sternberg, 2024). IQ tests typically measure intelligence based on cognitive ability; however, giftedness can take many forms (Subotnik et al., 2011). Occasionally, students’ performance on IQ tests can be impacted by test anxiety and lack of interest (Schillinger et al., 2021), deceiving them about their true abilities. Moreover, giftedness is a dynamic and evolving characteristic, not a fixed trait (Lo et al., 2019; Ziegler and Stoeger, 2017). A person’s cultural, linguistic, and socioeconomic background can also influence their performance on IQ tests (Holden and Tanenbaum, 2023). IQ tests are valuable for assessing cognitive ability and identifying gifted individuals. However, they have limitations and potential biases. Bias refers to systematic errors or unfairness in test design or administration that can disadvantage or advantage certain groups. IQ tests should be used alongside other measures to ensure a comprehensive evaluation of students’ strengths, reducing the impact of biases and providing a more accurate understanding of cognitive abilities. IQ tests can illuminate the path toward recognizing students with truly remarkable potential when used alongside a constellation of other assessments, such as creativity tests, academic performance, and observations by teachers (Abdulla Alabbasi et al., 2024a, 2024b; Abdulla Alabbasi et al., 2021; McBee et al., 2014; Renzulli and Reis, 2012; Runco et al., 2023).

Typically, IQ tests are constructed in accordance with intelligence theories such as Spearman (1904), Cattell (1963), and Cattell-Horn-Carroll theory (Schneider and McGrew, 2018). Cattell (1987) distinguished between fluid intelligence (Gf) and crystallized intelligence (Gc) as two distinct components of general intelligence (*g*). The Cattell-Horn-Carroll (CHC) theory combines two earlier theories: Cattell’s theory of fluid and crystallized intelligence and Horn’s theory of multiple intelligences (Carroll, 1993; McGrew, 2005). The fluid intelligence theory could be viewed as an integral part of the three main theories of intelligence. Spearman suggested that *g* contributes to performance across various intellectual tasks (Weiten, 2013). In this context, fluid intelligence can be viewed as a manifestation of general intelligence. Fluid intelligence reflects the ability to think rationally, solve problems, and think abstractly (Cattell, 1987). Accordingly, fluid intelligence aligns with Spearman’s concept of a general intelligence factor that affects various cognitive abilities. Moreover, fluid intelligence is a major part of the CHC theory (Schneider and McGrew, 2012). The framework of this theory considers fluid intelligence to be one of the broad factors that contribute to intelligence in general. It is a capability that allows one to adapt to novel situations and solve problems in an adaptive manner. As part of the CHC theory’s comprehensive model of intelligence, fluid intelligence is grouped together with other factors such as crystallized intelligence, visual–spatial processing, and working memory (Carroll, 1993). In these theories, fluid intelligence is recognized as a core component of intellectual functioning and as an integral component of various cognitive tasks and problem-solving situations.

However, it is important to point out that fluid factors may take other forms of human behavior. The monumental work of Terman and Oden (1959) on identifying high-IQ young people is well known, but he is also known in the research and evaluation literature for conducting one of the world’s most famous longitudinal studies. What was learned after following up on these subjects for almost 40 years? A detailed analysis was made of the 150 most successful and 150 least successful men among the gifted students in an attempt to identify some of the *non-intellectual factors* that affect success. Since the less successful subjects do not differ to any extent in intelligence as measured by tests, it is clear that notable achievement calls for a lot more than a higher order of intelligence. The follow-up study results indicated that personality factors are extremely important determinators of achievement.

### 1.1 The current study

Since IQ tests are culturally sensitive (Holden and Tanenbaum, 2023; Sattler, 2008), initiating and developing a local test that serves each country’s or culture’s needs is vital. The validity and reliability of any intelligence test are crucial to ensure that these tools are appropriate for any culture. Several intelligence tests have been translated and adapted into Arabic by the United Arab Emirates (UAE) and other Arab countries (Alfaiz et al., 2022). These tests include Stanford Binet (SB-5), Raven’s Progressive Matrices, Wechsler Nonverbal Scale of Ability, Wechsler Primary and Preschool Intelligence Scale, and Wechsler Intelligence Scale for Children-IV. Despite the UAE’s interest for more than two decades in identifying and developing services for gifted students in public and private schools, there was no scale or test that has been developed locally to be used in the identification of gifted students (AlGhawi, 2017; Daraghmeh, 2018). Translated intelligence tests are used in Arabic countries to identify gifted children, but they have some challenges and drawbacks. A major challenge of translation is the potential for linguistic and cultural biases (Laher and Cockcroft, 2017; van de Vijver and Tanzer, 2004). When intelligence tests are translated into Arabic, subtle semantic and cultural differences may affect their validity and fairness. In addition, the concept of giftedness itself may differ from culture to culture (Mandelman et al., 2010), as may the translation of tests to capture the cultural markers that are prevalent in Arabic societies. Furthermore, translating IQ tests to other languages could overlook sociocultural factors that can affect children’s performance (Shuttleworth-Edwards, 2016), such as educational disparities and limited resources. To ensure that gifted children receive assessment tools that are culturally and linguistically appropriate, it is essential to develop tools that are sensitive to their unique characteristics and experiences.

In order to overcome relying on translated intelligence tests, the Hamdan bin Rashid Al Maktoum Foundation for Medical and Educational Sciences (HF) has developed a complete assessment kit for identifying a range of student abilities, not just those who are gifted. This kit includes the first national intelligence test to be developed in the UAE, called the Hamdan Intelligence Scale (HIS), which is based on the CHC theory of intelligence (Carroll, 1993; Horn and Noll, 1997; McGrew, 2005; McGrew and Flanagan, 1998; Ziegler and Stoeger, 2016). The HIS intends to identify gifted students and evaluate intellectual abilities across the general population. This article uses Rasch theory and item response theory to evaluate the reliability and validity of the HIS in assessing students’ intellectual abilities in the UAE. This comprehensive evaluation is meant to validate the use of the HIS for a variety of educational and clinical applications beyond just the identification of gifted learners.

The single-parameter logistic, commonly known as the Rasch model, is a special case of Item response theory (IRT); it is considered a measurement rather than a statistical model. Rasch model has a long history in its application in the fields of social and behavioral sciences, including educational measurement (Hayat et al., 2020). The Rasch model yields ability estimates that are independent of the difficulty of the test and the ability of the other test takers. The model puts a person’s ability and item difficulty on the same scale, providing us with expectations about which items are most likely to be answered correctly by any given test-taker (Stemler and Naples, 2021). The true power of the Rasch model comes from its fit statistics, which allow us to evaluate whether or not we have truly built a linear scale that works the same way for all test takers, thereby facilitating meaningful interpretation of the test results. Under the Rasch model, test scores have a consistent meaning for all test takers in a way that they do not if one is using classical test theory.

## 2 Materials and methods

### 2.1 Participants

The participants in this study were 4,301 students: 1477 (34.3%) Male, and 2,824 (65.7%) Female from grade 4 to 6; 1,380 (32.1%) grade 4 (44.4% Male; 55.6% Female), 1,449 (33.7%) grade 5 (29.7% Male; 70.3% Female), and 1,472 (34.2%) grade 6 (29.3% Male; 70.7% Female). The sample has been selected from 22 elementary schools representing the seven Emirates of UAE: Abu Dhabi (34.7%), Ajman (2.0%), Dubai (20.2%), Fujairah (9.8%), Ras Al Khaimah (10.9%), Sharjah (11.8%), and Umm Al Quwain (10.6). The scale was administered during regular class time. Participation in the study was voluntary after obtaining consent from the participant’s parents. All participants were provided with profiles of their cognitive abilities as compensation for their cooperation. The ethical committee of the Ministry of Education approved the research.

### 2.2 Procedures

Data collection occurred from January 2022 to December 2022. Two separate rounds of sampling procedures were conducted to meet the sample size required to ensure the robustness of Rasch analysis results. Students were randomly selected from a list of students enrolled in the UAE government schools of Abu Dhabi, Ajman, Dubai, Fujairah, Ras Al Khaimah, Sharjah, and Umm Al Quwain. Parental consent forms and student assent forms were obtained from all participants. All students and their parents signed the consent forms. Exclusion criteria included students who did not obtain parental consent to participate in the study, students who were absent during the administration of the HIS, and students with identified learning disabilities or special education needs that could affect their performance on the HIS, as per school records.

### 2.3 Instruments

Hamdan Foundation has developed a complete kit for identifying gifted students. This kit includes the first national IQ test, the Hamdan Intelligence Scale (HIS), based on the CHC theory (Ziegler and Stoeger, 2016). HIS is a group-administered IQ test designed for students from grades 4 to 6. Students are presented with a series of 28 composite figures. Each figure contains three rows of figural elements. The figural elements’ progression across each row follows a certain construction rule. Students identify the construction rule by examining the first two rows and then applying the rule to correctly complete the incomplete final row (by choosing one out of four options) (Appendix 1). The HIS was administered through the Hamdan Bin Rashid Al Maktoum Foundation for Medical and Educational Sciences platform, which offers the HIS electronically (i.e., computer-based). The administration took place in schools’ computer labs and was monitored by Hamdan Foundation staff.

### 2.4 Data analysis

#### 2.4.1 Validity

The HIS test was designed to assess students’ general intelligence from grades 4 to 6. To verify the predicted factor structure of the HIS, we used parallel analysis to identify the number of factors that could be extracted according to the data. Then, the Confirmatory Factor Analysis (CFA) was conducted with LISREL (Version 8.8) software. These statistical methods were carried out on 4,301 students. CFA was used to ensure the constructive validity of the scales based on the previous literature (MacCallum and Austin, 2000) in order to test the internal structure of the data. It can be considered the most appropriate statistical framework that can be used to evaluate the validity and reliability of each item instead of the overall data, which allows the researchers to be able to design and adapt the scale (Joreskog and Sorbom, 2006). Initially, the “psych” R statistical package was run the parallel analysis method. Based on parallel analysis, it is suggested that one dimension can be extracted for the factor analysis, indicating that a single underlying factor explains the maximum variance in the data. A CFA was conducted to verify the hypothesized unidimensional model of HIS. It was carried out with structural equations following a maximum likelihood model. The results of the CFA of HIS provide support for the hypothesis that the test measures a unidimensional construct. This finding is evidenced by the factor loadings of the test items on a single general factor which ranged between (0.36–0.80; see Table 1). Furthermore, the fit indices of the HIS (χ^2^/df = 2.66, CFI = 0.94, GFI = 0.93, RMSEA = 0.069, and SRMSR = 0.065) indicate a good fit between the proposed theoretical model and the observed data.

**Table 1:** Means, standard deviations, and the results of confirmatory factor analysis of HIS.

Items	Mean	Standard deviation	Estimate	SE	z-value
Item_1	0.63	0.48	0.79	0.014	56.86**
Item_2	0.47	0.50	0.54	0.015	34.97**
Item_3	0.27	0.44	0.74	0.014	51.82**
Item_4	0.30	0.46	0.42	0.016	26.36**
Item_5	0.32	0.47	0.73	0.014	51.03**
Item_6	0.48	0.50	0.48	0.016	30.89**
Item_7	0.48	0.50	0.68	0.015	46.77**
Item_8	0.38	0.49	0.52	0.015	34.01**
Item_9	0.37	0.48	0.80	0.014	58.08**
Item_10	0.31	0.46	0.57	0.015	37.61**
Item_11	0.22	0.41	0.69	0.014	47.81**
Item_12	0.52	0.50	0.46	0.016	29.50**
Item_13	0.36	0.48	0.71	0.014	49.02**
Item_14	0.36	0.48	0.46	0.016	29.63**
Item_15	0.24	0.43	0.67	0.015	46.05**
Item_16	0.26	0.44	0.36	0.016	22.45**
Item_17	0.24	0.43	0.46	0.016	29.38**
Item_18	0.36	0.48	0.58	0.015	38.10**
Item_19	0.33	0.47	0.65	0.015	43.68**
Item_20	0.26	0.44	0.75	0.014	52.85**
Item_21	0.25	0.43	0.57	0.015	37.83**
Item_22	0.31	0.46	0.57	0.015	37.67**
Item_23	0.30	0.46	0.60	0.015	39.58**
Item_24	0.29	0.45	0.60	0.015	40.31**
Item_25	0.24	0.43	0.54	0.015	35.37**
Item_26	0.28	0.45	0.66	0.015	44.73**
Item_27	0.31	0.46	0.50	0.016	31.93**
Item_28	0.19	0.3 9	0.61	0.015	40.88**

**p < 0.01.

##### 2.4.1.1 Concurrent validity

The validity of the HIS was investigated using other IQ tests: (a) the Aurora-*g* Battery and (b) the Raven’s Standard Progressive Matrices.

###### 2.4.1.1.1 Aurora battery

The Aurora Battery is an assessment designed for children from 9 to 12 years of age. It is based on the theory of successful intelligence and one of its uses is for the identification of gifted students (Chart et al., 2008). The battery is composed of two parts: the first (Aurora-*g* Battery) measures general intelligence through series, analogy, and classification tests; the second (Aurora-*a* Battery) measures analytical, creative, and practical skills. The battery was translated and normed in Saudi Arabia (Aljughaiman and Ayoub, 2012). A total of 7,800 students were selected randomly from different areas that represent the Saudi Arabia. All the standardized loadings and their associated *t*-values for the Aurora_g, analytical, creative, and practical tests were significant. The fit indices for this full model were all excellent. Specifically, this model produced a nonsignificant χ^2^/df = 34.99, *p* = 0.069. In addition, the RMSEA = 0.048, GFI = 0.96, AGFI = 0.93, and NFI = 0.97 indicated the suggested model for Aurora fits with the data. The reliability coefficient of the Aurora-*g*, and Aurora-a by using Cronbach alpha were (0.86) for Aurora-g, (0.88) for analytical intelligence, (0.82) for creative intelligence, and (0.85) for practical intelligence. A sample of 357 students selected randomly from the UAE. The reliability coefficient of the Aurora-g was (0.82).

###### 2.4.1.1.2 Raven’s standard progressive matrices

A sample of 357 students selected randomly from the UAE. The reliability coefficient of the RSPM was (0.85).

Because the Aurora-*g* Battery and the RSPM were not normed in the UAE, only raw scores were used in correlational analyses. Correlations between the HIS and other scales were as follows: 0.83 with the Aurora-*g* battery and 0.86 with the Raven’s Standard Progressive Matrices.

##### 2.4.1.2 Developmental trends

Because intelligence grows rapidly in the early years (Chen and Siegler, 2000), age differentiation is used as a major criterion in the validation of intelligence tests. Intelligence test scores (raw scores) are expected to increase with advancing age (Anastasi and Urbina, 1997). Similar to age, years of schooling correlate with intelligence. In order to examine the developmental validity of the HIS, children’s raw scores in the norm sample were correlated with age and grade. The correlation between HIS and age was 0.79. As expected, the correlations between HIS and school achievement scores were 0.74, 0.76, and 0.81 for grades 4, 5, and 6. All correlation coefficients exceed 0.70.

#### 2.4.2 Reliability

Two types of reliability of the HIS were investigated. The internal consistency of scores was evaluated using Cronbach’s alpha for the entire sample and separately by grade level. The overall reliability coefficient for the full scale was 0.76. The reliability coefficients by grade level were 0.78, 0.75, and 0.73 for grades 4, 5, and 6, respectively. The test–retest reliability of the HIS was investigated to assess the consistency of the scores over a period of 4 weeks. The sample (*N* = 226) included children in grades 4 through 6. Correlations between the two administrations were corrected for attenuation (Murphy and Davishofer, 1988) was 0.89 for the full scale, and by grade level were 0.85, 0.88, and 0.86 for the grades 4, 5, and 6, respectively, which indicated strong evidence for the high reliability of the HIS scoring procedures.

## 3 Results

### 3.1 Descriptive statistics

In the descriptive analysis of the data collected for the 28 items, various statistical measures were calculated to provide a comprehensive understanding of the dataset. The mean values ranged from 0.19 to 0.63, indicating the average score for each item. The standard deviations, which measure the dispersion of the data, varied from 0.39 to 0.50, suggesting a little variation in standard deviation values across the scale items. The skewness of the item responses, which indicates the degree and direction of asymmetry, ranged from −0.55 to 1.57. Furthermore, the kurtosis values of the distribution ranged from −1.99 to 0.46 (see Table 1). The computed skewness and kurtosis values provide evidence that the distribution of data in the scale items closely approximates a Gaussian or normal distribution. The range of HIS test number-correct scores was quite similar across grade levels. The scores ranged from 0 to 24 for grade 4, 0 to 25 for grade 5, and 1 to 26 for grade 6. Also, the average test scores were 8.17, 9.27, and 10.20 for grades 4, 5, and 6, respectively. These descriptive statistics provide a preliminary understanding of the data distribution and will guide further inferential statistical analyses.

### 3.2 Unidimensionality and local independence of HIS items

To evaluate the unidimensionality of the 28-item HIS scale, a Principal Component Analysis of Standardized Residuals (PCASR) was conducted using WINSTEPS. The analysis revealed that the scale explained 14.8% of the total variance. Although this figure is below the commonly accepted threshold of 50% for confirming dimensionality (see Stolt et al., 2021), it is important to note that in Rasch analysis, unidimensionality is also supported by the eigenvalues of the first contrast being below 2, which was observed in this case. This finding aligns with previous Confirmatory Factor Analysis (CFA) results, which confirmed a one-factor model, further substantiating the scale’s unidimensional structure. Additionally, standardized residual correlations were examined to assess local independence, with the highest correlations found to be below 0.20, indicating that the items are locally independent (Christensen et al., 2017). Despite the lower variance explained, the combination of PCASR and residual correlation analysis provides evidence supporting the unidimensionality and local independence of the HIS scale.

### 3.3 Item fit to the Rasch model

The outfit and infit mean square values were examined to assess the fit of the items with the Rasch model. According to Linacre (2012, p. 444), “high infit mean squares indicate that the items are performing poorly for the targeted individuals.” This poses a greater threat to validity, although it is more challenging to be identified compared to high outfits. Good fitting items should ideally have infit and outfit mean-square values between 0.6 and 1.4 (Wright and Linacre, 1994). As indicated in Table 2, the infit and outfit mean-square values for the HIS items ranged from 0.88 to 1.14 and from 0.84 to 1.20, respectively. Overall, there were no significant indications of item misfit within the HIS scale. These findings suggest that our data align reasonably well with the Rasch model.

**Table 2:** Item difficulty, infit, and outfit statistics of the HIS scales’ items.

Items	Item statistics		
	*Diff*	*IN.MSQ*	*OUT.MSQ*
Item_1	−1.42	0.90	0.84
Item_2	−0.68	0.90	0.87
Item_3	0.30	1.03	1.02
Item_4	0.13	0.99	0.99
Item_5	0.04	0.96	0.95
Item_6	−0.70	0.88	0.86
Item_7	−0.72	0.97	0.96
Item_8	−0.27	0.92	0.89
Item_9	−0.21	0.95	0.95
Item_10	0.11	0.95	0.96
Item_11	0.62	1.04	1.12
Item_12	−0.90	1.01	1.01
Item_13	−0.14	0.92	0.91
Item_14	−0.17	1.02	1.05
Item_15	0.49	0.97	0.98
Item_16	0.39	1.01	1.03
Item_17	0.46	1.04	1.08
Item_18	−0.18	0.97	0.97
Item_19	0.01	1.05	1.06
Item_20	0.34	1.06	1.06
Item_21	0.45	1.14	1.20
Item_22	0.08	1.04	1.03
Item_23	0.13	1.04	1.04
Item_24	0.19	1.09	1.10
Item_25	0.46	1.02	1.06
Item_26	0.26	1.06	1.10
Item_27	0.10	1.06	1.09
Item_28	0.81	1.08	1.14

### 3.4 Person and item reliability using the Rasch model

The measurement properties of the HIS scale items were assessed using the separation index and reliability measures. The separation index for persons was found to be 1.27, indicating moderate measurement precision in distinguishing between individuals with varying levels of the measured trait. The separation index for items yielded a value of 13.04, suggesting a high level of measurement precision in differentiating between items with different levels of difficulty or severity (Wright and Stone, 1979). The Rasch reliability analysis revealed a reliability value of 0.62 for persons, indicating moderate internal consistency within the measurement scale (Lee et al., 2023; Linacre, 1994; Nunnally and Bernstein, 1994; Wright and Masters, 1982). Although falling below the desired threshold of 0.7, this value suggests a reasonable level of reliability, indicating some consistency in measuring the underlying trait among respondents. According to Linacre (1997), Cronbach’s alpha often overestimates reliability, inflating the coefficient, whereas Rasch person reliability provides a more conservative estimate. This suggests that Cronbach’s alpha value, though indicating acceptable reliability, may be inflated and should be interpreted with caution. Conversely, the reliability for items yielded a value of 0.99, indicating a high level of consistency in item difficulty or severity across the measurement scale (Wright and Masters, 1982).

### 3.5 Person and item calibration

**Figure 1:**
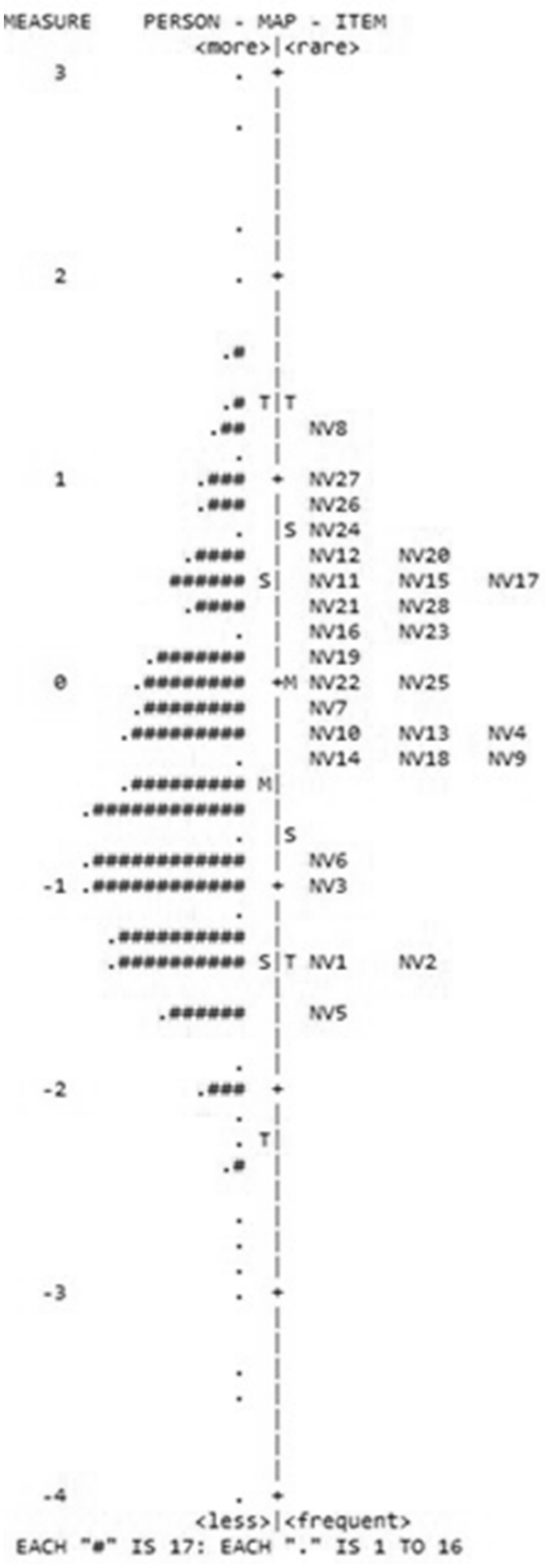
Person-item map for the HIS. Participants were represented on the left of the dashed line by the symbol “#.” On the right of dashed line were illustrated the items of each scale and their numbers. Higher ability for participants and more difficult items were on the top of the figure.

Table 3 displays the logit-based item difficulty parameters and the mean-square values for outfit and infit for each item. The item difficulty estimates in Table 2 varied from −1.42 to 0.81 logits, indicating a satisfactory range of item difficulty. Respondents found Item 28 to be the most challenging, while Item 1 was the easiest for them to endorse. Figure 1 shows the Person-item map for the HIS.

**Table 3:** Conversion table from raw sum scores of the NV to Rasch interval scores and the magnitude of the information.

*SCORE*	*Θ*	*S.E.*	*Info.*
0	−4.67	1.84	0.30
1	−3.43	1.03	0.95
2	−2.68	0.74	1.81
3	−2.22	0.62	2.59
4	−1.88	0.55	3.29
5	−1.60	0.51	3.92
6	−1.37	0.47	4.48
7	−1.16	0.45	4.97
8	−0.96	0.43	5.40
9	−0.78	0.42	5.77
10	−0.61	0.41	6.06
11	−0.45	0.40	6.30
12	−0.30	0.39	6.47
13	−0.14	0.39	6.58
14	0.01	0.39	6.62
15	0.16	0.39	6.60
16	0.31	0.39	6.51
17	0.47	0.40	6.36
18	0.63	0.40	6.14
19	0.79	0.41	5.85
20	0.97	0.43	5.50
21	1.16	0.44	5.07
22	1.37	0.47	4.57
23	1.60	0.50	4.00
24	1.87	0.55	3.35
25	2.21	0.62	2.63
26	2.66	0.74	1.84
27	3.39	1.02	0.96
28	4.63	1.84	0.30

“θ” refers to the Rasch interval scores, “SE” refers to the standard error of estimation. “Info.” refers to the magnitude of information at each score.

## 4 Discussion

This is the first study that uses both the CFA and Rasch analysis to validate HIS in upper elementary grades in the United Arab Emirates. The CFA was first conducted to verify the fitness of the one-factor model of the HIS (Mansolf and Reise, 2016). The current study utilized a common fitness index that was used by many other studies: the ratio of the chi-square statistic to the respective degrees of freedom (χ^2^/df), Comparative Fit Index (CFI), Goodness of Fit Index (GFI), Root Means Square Error of Approximation (RMSEA), and Standardized Root Mean Square Residual (SRMSR). A model fit was indicated by using a set of cutoff values: the CFI > 0.95, TLI > 0.95, RMSEA <0.06, and SRMSR <0.08 (Kline, 2016). The results of validity showed strong correlation coefficients between the Hamdan HIS and other scales such as Aurora-*g* battery and RSPM. Also, the results of the developmental trends demonstrated that raw scores of the HIS increase with age and grade relatively constantly across composite scores, providing developmental evidence for the validity of the HIS (Chen and Siegler, 2000).

The Rasch analysis was conducted to examine the unidimensionality of the HIS using a Rasch dichotomous model in the WINSTEPS computer program. The infit and outfit statistics were applied to verify whether item responses fit the expectations of the unidimensional Rasch model or not. An item with an infit or outfit mean square < 0.5 or > 1.5 demonstrated fit (Arisanti et al., 2020; Brown et al., 2016). Table 2 shows that all items fit and indicate unidimensionality. Principal component analysis (PCA) on residuals was applied to ascertain the unidimensionality further. According to Table 4, the residual variance of the first principal component was ≤20%, demonstrating unidimensionality (Wu et al., 2019). Rasch person reliability was analyzed for the items which fitted the unidimensional Rasch model. The criteria of Rasch person reliability were 0.62, acceptable reliability (Aaronson et al., 2002; Orji et al., 2012).

**Table 4:** Results of principal component analysis of the standardized residuals for the scale.

Samples	Unexplained variance			Separation index		Reliability	
	Total	First Contrast	Disattenuated Correlation	Persons	Items	Persons	Items
First	28 (85.2%)	1.7 (5.1%)	0.80	1.27	13.04	0.62	0.99

The results of person and item reliability using the Rasch model showed that the HIS scale items demonstrated moderate measurement precision for individuals while exhibiting high discrimination among items. The reliability analysis revealed moderate internal consistency for persons and excellent consistency in item difficulty or severity. In light of the previously mentioned, these findings support the reliability and effectiveness of the HIS scale in measuring the targeted traits. Moreover, the conversion table, which shows the conversion from HIS raw score to Rasch ability, could be useful for diagnostic purposes, as when considering reporting changes of the measured variable, an equal interval scaling allows the detection of any variations (see Table 3). In addition, by using this conversion table, the users do not need to conduct Rasch analysis every time to get the Rasch score when applying the HIS to assess intelligence levels in children.

## 5 Conclusion

The study provided evidence that the construct of the HIS displayed acceptable validity and reliability. Therefore, the findings obtained in this study provided strong support for the HIS in identifying gifted children in the UAE. This scale holds significant utility in assessing upper elementary grade students’ cognitive abilities from grades 4 to 6. The validity evidence presented in this study strongly supports the use of the HIS for its intended purposes. The strong correlation coefficients between the HIS and established measures such as the Aurora-*g* Battery (0.83) and the Raven’s Standard Progressive Matrices (0.86) demonstrate the scale’s convergent validity. This indicates that the HIS is effectively measuring the same underlying construct of intelligence as these well-recognized instruments. Furthermore, the developmental trends observed in the study provide further validation for the HIS. The consistent increase in raw scores on the HIS with age and grade level suggests that the scale accurately captures the target population’s expected cognitive growth and development. This lends support to the scale’s ability to differentiate between students of different ages and grade levels, which is a crucial aspect of an intelligence assessment tool. The Rasch model analysis conducted in the study also reinforces the psychometric soundness of the HIS. The acceptable range of infit and outfit mean squares and the low eigenvalues of the first contrast confirm the scale’s unidimensionality. This means that the HIS is effectively measuring a single, coherent construct of intelligence, as intended. Additionally, the Rasch person reliability result of 0.62 falls within the acceptable range, further supporting the reliability of the HIS in the UAE context. In conclusion, the robust validity and reliability evidence presented in this study strongly supports the use of the Hamdan Intelligence Scale (HIS) for assessing the cognitive abilities of upper elementary grade students within the United Arab Emirates. The scale’s development and standardization within the local context and its demonstrated psychometric properties make it a valuable tool for educators, researchers, and clinicians working with this population.

Validation of test scores involves evaluating the plausibility of claims based on those scores, as Kane (2013) outlined. An argument-based approach to validation requires presenting the claims as an argument, specifying inferences, and supporting assumptions. This process entails assessing the coherence, completeness, and plausibility of the interpretation/use argument. Key points emphasized by Kane (2013) are as follows: First, the validation focuses on the proposed score interpretations and uses rather than the test or scores themselves. Second, the validity of interpretations or uses relies on the strength of supporting evidence. Third, more ambitious claims require stronger support and pose greater validation challenges. Fourth, interpretations and uses can evolve over time, necessitating adjustments in the evidence required for validation. According to Kane (2013), the researchers employed multiple methods to assess the validity of the current test. Concurrent validity was examined using two IQ tests, namely the Aurora-*g* Battery and the Raven’s Standard Progressive Matrices. The constructive validity of the HIS was investigated through Confirmatory Factor Analysis (CFA) and the dimensionality was tested using Principal Component Analysis of the standardized residuals (PCASR) to confirm the unidimensionality of the HIS 28-item scale. Additionally, the researchers examined the developmental validity of the HIS by correlating children’s raw scores in the norm sample with their age and grade. These rigorous validation methods provide substantial evidence for the validity of the current test.

Future research will involve piloting items with varying levels of difficulty and analyzing their fit using the Rasch model to ensure they appropriately target high-ability individuals. It will continue to investigate the HIS further to examine its predictive validity in the long run. In addition, more studies are required to study the ability of the HIS to measure differentiation among diverse and different groups, such as gifted and average children, students with learning difficulties, low achievers, and low-income and high-income environments (Ayoub et al., 2021). In addition, suggest longitudinal studies to track the HIS’s effectiveness over time and studies to explore its application beyond the UAE to other Arabic-speaking regions.

## 6 Limitations

The current work has some limitations. First, it has a relatively small sample size compared to the UAE population and the study is exploratory in nature. Moreover, future research needs to consider more gender-balanced sampling. In the current work, girls were overrepresented, although gender differences in some cognitive abilities are negligible (Abdulla Alabbasi et al., 2022; Giofrè et al., 2022), including IQ. Third, the HIS only assesses fluid intelligence; thus, it might not be a choice when it comes to assessing crystallized/learned intelligence. Additionally, as indicated in Table 2, the scale lacks difficult items, which may limit its ability to differentiate among higher-ability participants. Future studies should focus on developing more challenging items to better discriminate among individuals with higher abilities. Finally, the HIS is only valid for administering to UAE students. Future research might test the validity and reliability of the HIS in other cultures.

## Data availability statement

The raw data supporting the conclusions of this article will be made available by the authors, without undue reservation.

## Ethics statement

The studies involving humans were approved by Ethical committee, Ministry of Education, UAE. The studies were conducted in accordance with the local legislation and institutional requirements. Written informed consent for participation in this study was provided by the participants’ legal guardians/next of kin.

## Author contributions

AAA: Conceptualization, Formal analysis, Software, Visualization, Writing – review & editing. AMA: Resources, Writing – original draft. MA: Funding acquisition, Project administration, Writing – review & editing. AM: Data curation, Project administration, Writing – review & editing. EO: Methodology, Software, Writing – review & editing. AMAA: Methodology, Writing – review & editing. JR: Validation, Writing – review & editing.

## Figures and Tables

**Figure A1:**
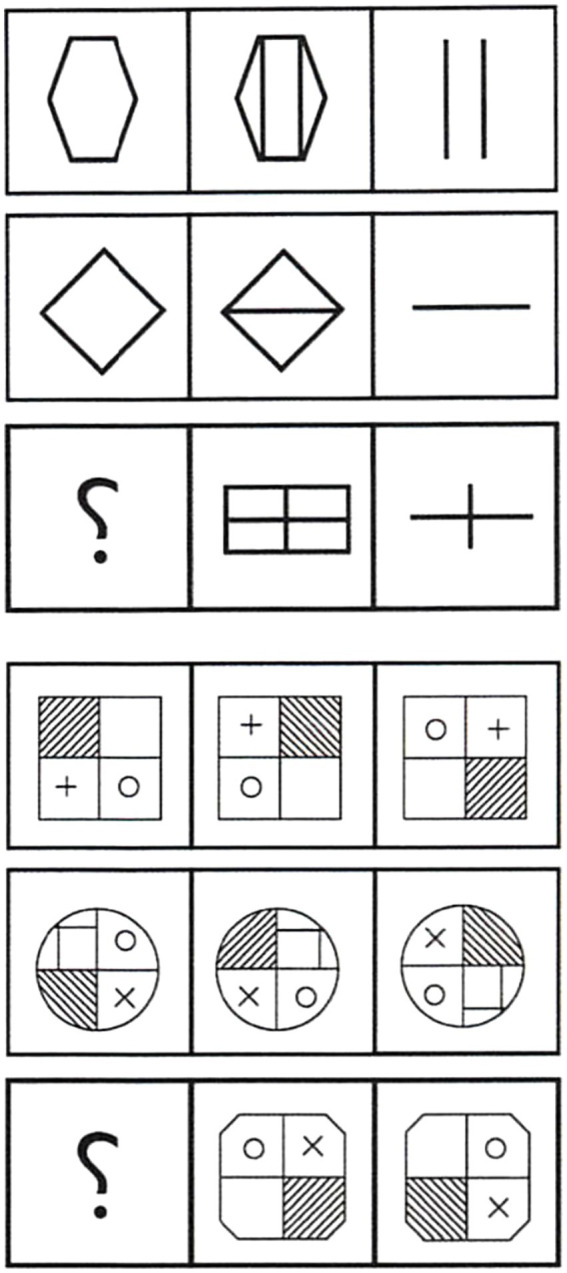
Examples of the HIS.
